# Monitoring of Ochratoxin A Occurrence and Dietary Intake in Tarhana, a Fermented Cereal-Based Product

**DOI:** 10.3390/foods14030443

**Published:** 2025-01-29

**Authors:** Esra Akkaya, Meryem Akhan, Burcu Cakmak Sancar, Hamparsun Hampikyan, Ayse Seray Engin, Omer Cetin, Enver Baris Bingol, Hilal Colak

**Affiliations:** 1 Department of Food Hygiene and Technology, Faculty of Veterinary Medicine, İstanbul University-Cerrahpaşa, Istanbul 34500, Türkiye; bingolb@iuc.edu.tr (E.B.B.); hcolak@iuc.edu.tr (H.C.); 2 Department of Nutrition and Dietetics, Faculty of Health Sciences, Istanbul Esenyurt University, Istanbul 34510, Türkiye; meryemakhan@esenyurt.edu.tr (M.A.); burcucakmak@esenyurt.edu.tr (B.C.S.); 3 Department of Gastronomy and Culinary Arts, Faculty of Fine Arts, Istanbul Beykent University, Istanbul 34500, Türkiye; hamparsun@beykent.edu.tr; 4 Department of Gastronomy and Culinary Arts, Faculty of Fine Arts, Istanbul Gelisim University, Istanbul 34310, Türkiye; ascetin@gelisim.edu.tr; 5 Department of Nutrition and Dietetics, Faculty of Health Sciences, Istanbul Rumeli University, Istanbul 34570, Türkiye; omer.cetin@rumeli.edu.tr

**Keywords:** ochratoxin A, tarhana, cereal-based product, mycotoxin contamination, dietary intake, traditional food

## Abstract

The aim of this study was to determine the mold and ochratoxin A (OTA) contamination of tarhana, a traditional product widely consumed in Turkish cuisine. For this purpose, a total of 350 tarhana samples (homemade and industrially produced) were randomly collected from retail stores, markets, and bazaars in different regions of Türkiye and analyzed by means of LC-MS/MS for the occurrence of OTA. According to the results, OTA was detected in 36 of 150 (24%) industrially produced tarhana samples, with a concentration range of 0.12–2.34 µg/kg, while 118 of 200 (59%) homemade tarhana samples contained OTA, with the range from 0.16 to 4.15 µg/kg. Only 8 of 350 (4%) homemade tarhana samples were found to be above the maximum permissible limit (3.0 µg/kg) for OTA. The mold contamination was found to be higher in homemade tarhana (3.756 log CFU/g) than in the industrially produced samples (2.742 log CFU/g). The estimated weekly intake values of OTA with tarhana consumption were well below the provisional tolerable weekly intake values for both industrially produced and homemade tarhana samples, even when consumed every day of the week, indicating that dietary intake of OTA through tarhana consumption does not pose a health risk. In conclusion, optimizing the fermentation and drying conditions applied during tarhana production and ensuring proper hygiene conditions can help to reduce the risk of OTA contamination. Moreover, monitoring and testing the OTA levels in tarhana on a regular basis can also ensure the food safety of this product.

## 1. Introduction

Mycotoxins produced by molds are toxic compounds that can potentially contaminate food and feed, causing serious public health risks [1]. Ochratoxin A (OTA), a common mycotoxin that is produced by certain mold species, particularly by *Aspergillus* and *Penicillium* species, can adversely affect food safety and public health by contaminating a variety of food products, including cereals, coffee, wine, and dried fruits [2,3,4]. Ochratoxin A is classified as a probable carcinogen (Group 2B) by the International Agency for Research on Cancer (IARC) and is also considered as nephrotoxic, having caused kidney toxicity (nephrotoxicity) and kidney tumors in animal studies [5]. Due to its toxic effects, OTA is subjected to regulations and maximum permitted levels in food and feed worldwide. These regulations aim to protect public health by limiting exposure to OTA through consumption. Maximum levels vary depending on the specific food or feed commodity and the jurisdiction [6,7]. Therefore, the Turkish Food Codex has established the maximum limit of OTA as 3.0 μg/kg in cereals and cereal products [8], similar to the Commission Regulation (EU) 1881/2006, which set the limit of OTA at 3.0 μg/kg in cereal-based food products [9], whereas the Food and Drug Analysis (FDA) has not reported a regulation on the presence of OTA in food [10]. The determination of OTA concentrations in foods is critical for the assessment of dietary exposure levels and the associated adverse health effects on public health. In this regard, the provisional tolerable weekly intake (PTWI), which indicates the level of OTA that can be consumed on a weekly basis without posing a health risk, is an indispensable component in the assessment of food safety [11]. The PTWI of OTA was set at 100 ng/kg b.w./week by the Joint FAO/WHO Committee of Experts on Food Additives [12], while the tolerable weekly intake (TWI) of OTA was published as 120 ng/kg b.w./week by the EFSA CONTAM Panel [13]. Moreover, the EFSA [14] stated that due to the increased uncertainty in the mode of action of renal carcinogenicity in recent studies, it is not appropriate to set a health-based guideline value, implementing a margin of exposure (MOE) approach. Hence, OTA contamination, an important food safety issue due to its widespread occurrence, toxicity, and potential health risks, can be prevented through good agricultural and good manufacturing practices throughout the food production chain. At this point, regular monitoring and testing of mycotoxin levels and mold control are critical to ensure the sustainability of food safety [15,16].

Tarhana is a traditional fermented cereal-based product prepared by mixing cereal flour (wheat or barley), yoghurt, and a variety of vegetables and spices, followed by fermentation, drying (sun-drying or oven-drying), and grinding processes, and it is popular in various countries, particularly in the Mediterranean region, Middle East, and Central Asia [17,18,19,20]. A versatile and nutritious food product with a rich cultural heritage, tarhana is a good source of carbohydrates, protein, fiber, vitamins, and minerals. The fermentation process enhances the bioavailability of nutrients and may also increase the content of certain vitamins, such as B complex and vitamin A [21]. The nutritional benefits, unique flavor, and texture of tarhana make it popular in many cuisines around the world, especially in Türkiye [18,22].

Due to its ingredients and fermentation process, tarhana is a cereal-based product potentially susceptible to contamination of ochratoxin. Contamination of wheat flour, yoghurt, vegetables, and spices in tarhana with mold before processing can lead to ochratoxin occurrence [17,19]. In addition, the fermentation process can provide a favorable environment for the growth of mold under inappropriate conditions. Moreover, inconvenient storage conditions after production, such as high humidity levels or poor ventilation, can increase the risk of mold growth and thus ochratoxin contamination. Although exposure levels in studies examining OTA levels in cereals and cereal products are generally within the tolerable weekly intake (PTWI) limits set by the JECFA (100 ng/kg body weight per week), it has been emphasized that large quantities or chronic consumption of contaminated foods may exceed safe limits, making health risks possible. The carcinogenic, nephrotoxic, and immunotoxic effects of OTA raise concerns about its intake through food [23,24].

Various analytical techniques have been used for the accurate, sensitive, and selective detection and quantification of OTA in cereal-based products. Liquid chromatography–mass spectrometry (LC-MS/MS) is one of the analytical methods utilized for the determination of OTA in tarhana. The high accuracy of this method allows for the detection of even very low concentrations of OTA [25,26].

Studies from several countries have documented the presence of mycotoxins, including OTA in cereal-based products [2,17,27,28,29,30,31,32,33]. However, there are limited studies revealing the occurrence of OTA in tarhana, a fermented cereal-based product. Therefore, this study aimed to determine the mold and ochratoxin A contamination in tarhana, a traditional product widely consumed in Middle Eastern, Mediterranean, and Central Asian cuisines, including Turkish cuisine.

## 2. Materials and Methods

### 2.1. Sampling

A total of 350 tarhana samples, including homemade (200) and industrially produced (150) tarhana, were randomly collected from retail stores, markets, and bazaars from September 2021 to February 2024 (samples were collected between the September and February of each year for three consecutive years). The samples were collected in a plastic bag (at least 500 g for each sample), stored at 4 °C for analysis within a week, and kept at room temperature for a maximum of 30 min prior to analysis.

### 2.2. Chemicals, Reagents, and Standards

All LC solvents (LC–MS grade, 99.9%) and other chemicals were acquired from Merck (Darmstadt, Germany). OTA was obtained from Sigma-Aldrich (St Louis, MO, USA), and the stock solution of OTA was prepared as 1 mg/mL in methanol. A solution of ^13^C-labeled OTA in acetonitrile (~10 μg/mL) was used as an internal standard (Sigma-Aldrich, St Louis, MO, USA).

### 2.3. Extraction Procedure

The extraction procedure for OTA in tarhana samples was carried out according to the direction described by Meerpoel et al. [25]. Briefly, 4 g of homogenized samples were extracted with 10 mL of 10% acidified saline solution (*m*/*v*); afterwards, 20 mL of extraction mixture containing acetonitrile and acetic acid glacial (99:1, *v*:*v*), and 10 mL of hexane were added to tarhana samples. The mixture was shaken at 22 ± 2 °C for 60 min, and then magnesium sulphate (6.0 g) and NaCl (1.5 g) were added, followed by shaking to thoroughly disperse the salts in the mixture. A total of 1 mL of the supernatant from samples centrifuged at 4000× *g* for 5 min was evaporated to dryness under a stream of nitrogen. The dried material was dissolved in an injection solvent (methanol–ultrapure water, 1:1, *v*:*v*) then vortexed and centrifuged (5000× *g*, 10 min). A 5 µL of ^13^C-labeled internal standard solution (100 ng/mL) was added to 45 µL of extract, and 5 µL of the mixture was injected into the LC–MS system.

### 2.4. LC-MS/MS Analysis

LC-MS/MS analysis was conducted in accordance with the procedure provided by Meerpoel et al. [25] with Agilent 6460 Triple Quadrupole LC/MS systems (Agilent Technologies, St. Clara, CA, USA). Chromatographic separation was carried out using an Agilent C18 column (Particle size: 1.8 μm; 2.1 mm × 100 mm). The column temperature was 40 °C. The gradient elution was executed with a mobile phase consisting of eluent A (0.05% acetic acid, *v*/*v*, and 5 mM ammonium acetate in water) and eluent B (methanol) at a flow rate of 0.4 mL/min. The gradient started at 30.0% and progressed to 90% over 6.5 min, with an increase in B. This proportion of B was increased to 100% over 9 min, and the run time was completed in 10 min in total. MS analysis was conducted using multiple reaction monitoring (MRM) with positive (OTA) electrospray ionisation (ESI+/−). The optimum values applied for LC-MS/MS conditions (retention time, cone voltage, collision energy, precursor and precursor ions) were 4.6 min, 35 V, 238.9 *m*/*z*, and 404.0 *m*/*z*, respectively.

### 2.5. Recovery Evaluation

Recovery studies were performed by spiking blank samples that were fortified with OTA standard at concentrations ranging from 0.1 to 10 µg/kg. The spiked samples were left for 15 min equilibration, and the OTA levels were determined according to the previously described procedure. The method showed good recoveries that were in the range of 89–97% with residual standard deviation varying from 3.6% to 6.9%. The limit of detections (LOD) and limit of quantifications (LOQ) were 0.5 and 1.0 µg/kg, respectively.

### 2.6. The Dietary Intake Assessment

The dietary intake of ochratoxin A was calculated on the basis of the average contamination level and the average consumption data of the food via the following equation [34,35].

The estimated weekly intake (EWI—g/kg b.w./week) = (the average concentration of OTA in food [C_OTA_] × the average consumption of tarhana [K_T_])/body weight (kg)

According to the report of the Turkish Nutrition and Health Survey [36], the frequency of tarhana consumption varies in Türkiye. Therefore, in this study, food intake values were calculated considering different consumption frequencies, i.e., 1, 3, 5, and 7 times a week (since there are no exact data on the consumption of tarhana per capita). For each exposure fact, the dietary intake of OTA was assessed following the consumption of tarhana (as a soup) served as 20 g for one serving or portion. Body weight is based on 70 kg, which is the average body weight for the adult population recommended by EFSA [37].

The estimated weekly intakes calculated by the above-mentioned formula were compared with the provisional tolerable weekly intake (PTWI) established by JECFA [12] to assess the health effects of OTA exposure through consumption of cereal-based products. PTWI values represent safe permissible levels for OTA levels in tarhana samples. The fact that the EWI is lower than the PTWI indicates that OTA exposure by consumption of tarhana does not pose a health risk.

A risk assessment for neoplastic and non-neoplastic effects of OTA was also performed using a margin of exposure (MOE) approach recommended by EFSA [14], with the following equation.

Margin of exposure (MOE) = BMDL_10_ (benchmark dose lower bound)/EDI (estimated daily intake) (1)

For non-neoplastic effects, a BMDL_10_ value of 4.73 μg OTA/kg b.w./day was used based on the effect of causing kidney lesion in pigs. An MOE value of more than 200 is considered to be of low health concern. For chronic neoplastic effects, a BMDL_10_ value of 14.5 μg OTA/kg b.w./day was used based on the effect of leading to the formation of kidney tumors in rats. An MOE greater than 10,000 indicates a low concern for health.

### 2.7. Physicochemical and Microbiological Examination

The pH value of tarhana samples was recorded using a digital calibrated pH meter (Hanna HI-9321, Woonsocket, RI, USA) at room temperature. The water activity (a_w_) of samples was measured with an aw meter (Decagon AquaLab LITE, Washington, DC, USA), and the moisture content of tarhana samples was assessed via the method of AOAC [38], which involves drying a homogeneous mixture at 105 ± 2 °C to a constant weight.

For microbiological analyses, 25 g of tarhana from each sample was homogenized by mixing with 225 mL sterile peptone water [39]. Serial dilutions were inoculated on Dichloran rose Bengal chloramphenicol (DRBC) agar (Oxoid, CM1148) supplemented with reconstitute chloramphenicol (SR0078E) and incubated at 25 °C for 5 days for the determination of mold counts in tarhana samples [40]. The mold count was expressed as log CFU/g, and the counted mold colonies were macroscopically evaluated on a species basis after subculturing in potato dextrose agar (Oxoid, CM0139) incubated at 25 °C for 2–4 days in order to obtain a pure culture. The colonies were determined according to their distinct morphological characteristics, such as colony shape, size, texture, and color.

### 2.8. Statistical Analysis

Analysis of variance (ANOVA) of SPSS 21.0 (SPSS Inc., Chicago, IL, USA) was performed to evaluate the differences between the mean OTA concentrations of the tarhana samples, and Duncan’s multiple range test was used to determine the significance of differences (*p* < 0.05). Pearson’s correlation coefficients (r) were used to assess the correlation between the OTA levels, mold counts, and physicochemical parameters of tarhana samples.

## 3. Results and Discussion

**Figure 1:**
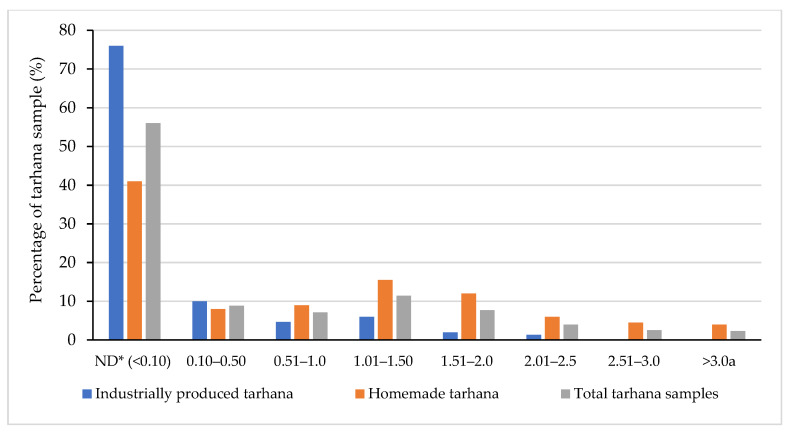
The detection rate of OTA in tarhana samples (*n* = 350). * ND: not detected (detection limit: 0.10 μg/kg). ^a^: The maximum permissible limit of OTA set by the Turkish Food Codex and European Commission.

Occurrence of OTA in tarhana samples is shown in Table 1. A total of 154 out of 350 tarhana samples were found to be above the detection limit value. A total of 36 out of 150 (24%) industrially produced tarhana samples contained OTA, while none of these samples exceeded the maximum limit of the Turkish Food Codex (TFC) Regulation on Contaminants and the Commission Regulation (EU) 1881/2006. OTA was detected in 118 of the 200 (59%) homemade tarhana samples, and 8 out of 118 (4%) samples exceeded the maximum regulatory limit of 3.0 µg/kg according to the TFC [8]. About 76% of the industrially produced tarhana samples and 41% of the homemade tarhana samples were found to be below the detection limit of 0.10 μg/kg (Figure 1).

The OTA levels of the tarhana samples were categorized for each concentration range from 0.50 μg/kg up to the maximum limit value of 3.0 μg/kg to demonstrate the distribution of the OTA levels detected in the tarhana samples. The difference between the mean OTA values of the samples separated according to the detection levels was found to be significant (Table 1; *p* < 0.001). A total of 56 out of 350 tarhana samples had OTA concentrations below 1.0 μg/kg, while 67 samples had OTA concentrations between 1.0 and 2.0 μg/kg, and 23 samples had OTA concentrations between 2.0 and 3.0 μg/kg. An OTA concentration above 3.0 μg/kg was determined in eight of the samples, and the mean value was determined to be 3.646 ± 0.287 μg/kg.

The industrially produced tarhana samples contained OTA in the range of 0.12–2.34 µg/kg, with the mean level of 0.21 ± 0.64 µg/kg, which is quite below the maximum limit of 3 μg/kg for cereal product set by the TFC [8] and European Commission [9]. On the other hand, OTA concentrations in the homemade tarhana samples were found to be in the range of 0.16–4.15 µg/kg. Although the maximum level detected in the homemade tarhana samples was slightly above the regulated limit, the mean level (0.93 ± 0.90 μg/kg) was below 3 μg/kg. It was determined that only 2.29% of the industrial and homemade tarhana samples exceeded the limit value in terms of OTA concentration (Table 2).

There have been a few studies reporting the occurrence of OTA in tarhana samples from Türkiye. The incidence of OTA in tarhana detected in the present study was low (154/350 samples, 44%) compared to that in Kaymak et al. [19] and Ozden et al. [17]. Kaymak et al. [19] reported that 32 out of 53 (60.38%) tarhana samples contained OTA at concentrations ranging from 0.1 to 6.7 μg/kg. The average OTA content of 29 out of 32 samples was in the range of 0.1–1.0 μg/kg; 2 of them were in the range of 1.0–5.0 μg/kg; and 1 of them was in the range of 5.0–20.0 μg/kg. It was stated that only one of the tarhana samples exceeded the TFC and EU maximum limit of 3.0 μg/kg, with an OTA content of 6.7 μg/kg. On the other hand, Ozden et al. [17] found that 47 out of the 84 (55.95%) tarhana samples analyzed contained OTA, and the mean concentration of contaminated samples was recorded as 0.41 μg/kg, which is below the limit value authorized by the European regulation. The raw materials used in the production of tarhana may be contaminated with mycotoxins, which may also be found in the final product due to their thermostability. Moreover, inadequate drying and/or inappropriate storage conditions during the tarhana production process are also responsible for the formation of OTAs [2,17,26]. In the present study, the higher prevalence of OTA in homemade tarhana samples compared to industrially produced tarhana was generally associated with the production conditions. Uncontrolled fermentation process, inappropriate drying conditions (open air and/or inadequate drying), and insufficient storage conditions (storage of both the wet dough obtained by mixing the ingredients and the dry dough/product after drying) applied during homemade production have a direct effect on OTA contamination in the final product.

The raw material, which mainly affects the presence of OTA in the final product, also determines the concentration of OTA in tarhana. Wheat flour, which is an essential component of many foods such as bread, pasta, and crackers, is also one of the main raw materials used in tarhana production. While the low water activity of flour enables it to be microbiologically safe, it does not prevent the survival of molds. Changes of 1% or 2% in the moisture content of flour promote mold growth and OTA formation [2,41]. The prevalence and concentration of mycotoxins in cereals may also affect the level in processed products prepared from these cereals. In this respect, flour contaminated with mycotoxin can be a major source of contamination for cereal-based products [42].

Cereal-based food has been identified by the European Commission as the primary contributors to human exposure to OTA [43]. In a report submitted by EU member states, the contribution of dietary cereals to OTA exposure was estimated to be 44% [44]. It was emphasized by Li et al. [45] that this is in line with the assessment presented by the EFSA CONTAM Panel stating that cereals are the primary source [14]. In the meta-analysis conducted by Khaneghah et al. [42], OTA was identified as the mycotoxin with the highest prevalence in cereals, and the prevalence of OTA in cereal foods was reported as 51%.

Demirel and Sariozlu [46] reported an incidence of OTA contamination of 84% in grain-based flour samples, and the mean OTA levels ranged between 0.80 and 4.76 μg/kg. The authors also noted that two of these samples (3.02 μg/kg and 4.76 μg/kg) exceeded the maximum limit of 3.0 μg/kg set by the EC. In another study, Kara et al. [47] analyzed 100 cereal flour samples (60 wheat, 24 maize, and 16 rice flour samples) for the occurrence of OTA and reported that the levels were well below the maximum limits specified in EU legislation. OTA was detected in 16 (26.7%) of the 60 wheat flour samples analyzed, while the range of values was reported to be between 0.105 and 0.918 μg/kg. Cüce [48] determined the OTA contents in wheat flours analyzed for three consecutive years to be 1.66 ± 0.27, 1.52 ± 0.28, and 1.63 ± 0.28 μg/kg for 2017, 2018, and 2019, respectively. The researcher emphasized the primary effect of weather variables such as humidity, precipitation, and temperature values on mycotoxin formation in foods and revealed that the highest mycotoxin formation was formed in the year with the highest humidity and temperature values in the three years surveyed. Similarly, Spahiu et al. [49] recorded that the OTA in flours (wheat, corn, and rye) was in the range of 4.9 and 14.8% (0.26–2.75 μg/kg).

Meanwhile, Hajok et al. [50] reported that the presence of OTA was detected in 13 (11.5%) out of 113 wheat flour samples from foods available on the market in Poland, and two of these samples exceeded the maximum permissible OTA level. Furthermore, it was noted that 2 of the 11 wheat grain samples analyzed were between the detection limit and the maximum permissible level of 5.0 μg/kg. The research conducted by Elaridi et al. [51] showed that 4 (8%) out of 50 wheat flour samples were contaminated with OTA at levels ranging from 0.6 to 3.4 μg/kg (mean 1.9 ± 0.2 μg/kg), and only one of the positive samples had a concentration above the maximum limit (3.0 μg/kg). On the other hand, wheat grain samples did not contain OTA in detectable levels. The researchers associated the detection of OTA in wheat flour samples, and not in wheat grains, with the humidity and high temperature to which wheat flours are exposed during both processing and storage stages. Thus, they emphasized that the effective drying process applied to foods such as cereals and cereal-based products is critical to minimize mycotoxin formation. Cereal grains have been shown to be the main source of daily OTA intake [52]. Kuruc et al. [53] notified OTA in 3.8 to 29.7% of cereal grain samples with levels of 0.16 to 185.2 μg/kg obtained from the United States. With a similar rate, Soleimany et al. [54] also reported an OTA incidence between 3 and 21% in cereal grains. Kirinčič et al. [55] determined OTA in cereal grains to have a level between 1.1 μg/kg and 6.7 μg/kg, while it was stated that this range was between 0 and 2.1 μg/kg in cereal-based food.

Kabak [27] reported that OTA was the most frequently detected mycotoxin, with a detection rate of 43.6% (48/110) in retail cereal products. However, none of the 48 samples containing OTA exceeded the limit value of 3.0 μg/kg, and OTA concentrations were found to be in the range of 0.066–1.125 μg/kg. The researcher concluded that the OTA contamination of retail cereal products is mostly associated with wheat-based cereals. Similarly, from Portugal, Assunção et al. [29] reported OTA in 50% of cereal-based products, with a mean level of 0.061 μg/kg. Higher rates (58%) were stated by Kumar et al. [56] in wheat samples from India (1.36 and 21.17 μg/kg). In another study concerning Mediterranean countries, Serrano et al. [57] detected OTA in wheat-based products at a rate of 3%.

In addition to wheat flour, which is the main ingredient in tarhana production, other ingredients, such as yoghurt or ayran, various vegetables/tomato paste, and spices (red pepper and salt), also involve mold contamination and thus the possibility of mycotoxin formation in the final product. The level of OTA in yoghurt and ayran—both fermented dairy products—depends primarily on the content of OTA in the milk used in their production [58]. Although the consumption amounts of spices are not as high as those of cereals and cereal-based products, they can also pose a serious risk of OTA contamination due to their processing and storage stages [31]. The maximum limit of OTA in spices was set by the EC [59] as 15–20 μg/kg. Ozbey and Kabak [60] detected OTA in 75% (18/24) of red chilli flakes, with concentrations ranging from 0.46 to 53.04 μg/kg, while 54.5% (12/22) of red chilli powder samples were had level ranges of 0.78–98.2 μg/kg. Red pepper is regarded as a product that is highly susceptible to fungal contamination and thus to mycotoxin formation due to both processing conditions (harvesting, drying, and storage) and environmental changes (temperature and humidity) [60]. Tosun and Ozden [61] reported the detection of OTA in 27 out of 31 packaged red pepper flakes, with levels ranging from 0.6 to 1 μg/kg. However, all 44 unpackaged red pepper samples analyzed contained OTA at concentrations of 1.1–31.7 μg/kg, of which 4 were above the limit value of 15 μg/kg. Gambacorta et al. [62] found that OTA contamination in fresh and processed sweet pepper samples was 51%, with a mean value of 29.5 μg/kg. This mycotoxin contamination in peppers was also confirmed by Wang et al. [63]. El Darra et al. [64] reported that 30% of spices and 11% of herbs sold in Lebanon were contaminated with OTA, and 10% of these samples exceeded European limits.

The estimated weekly intake (EWI) values of OTA with tarhana consumption are expressed in Table 3. The mean values of EWI for industrially produced tarhana samples were 0.06, 0.18, 0.30, and 0.42 ng/kg b.w./week for four different consumption frequencies (groups 1 to 4), which represents 0.1%, 0.2%, 0.3%, and 0.4% of the PTWI, respectively. Even at the highest weekly consumption of industrially produced tarhana, the estimated exposure was found to be considerably lower than the PTWI of 100 ng/kg bw/week set by JECFA. Meanwhile, for homemade tarhana samples, the mean values of EWI were found to be 0.27, 0.80, 1.33, and 1.86 ng/kg b.w./week for groups 1 to 4, which is equivalent to 0.3%, 0.8%, 1.3%, and 1.9% of the PTWI, respectively. In the group with the highest OTA exposure due to tarhana consumption (1.860 ng/kg b.w./week), there was an exposure representing 1.9% of the PTWI, which is approximately 53 times less than 100 ng/kg b.w./week. For a 70 kg adult, the PTWI limit is 7000 ng/week, and the maximum OTA intake with tarhana consumption would be 130 ng/week, which is well within the safe limit. Based on the estimated weekly intakes calculated with the OTA levels found in the tarhana samples in our study, the OTA exposure of adults who consume the highest amount of tarhana in Türkiye is well below the JECFA PTWI value of 100 ng/kg b.w./week, indicating that dietary intake of OTA through tarhana consumption does not pose a health risk.

Moreover, the margin of exposure (MOE) was calculated to determine the carcinogenic effects of OTA exposure (Table 4). The MOE values for the industrially produced tarhana samples determined from the estimated daily OTA intakes were 78.83 (average intake) and 26.28 (extreme intake) for the non-neoplastic effect and 241.67 (average intake) and 80.56 (extreme intake) for the neoplastic effect. The MOE values for the homemade tarhana samples were 17.78 (average intake) and 5.94 (extreme intake) for the non-neoplastic effect and 54.51 (average intake) and 18.19 (extreme intake) for the neoplastic effect. MOEs greater than 200 and 10,000 for non-neoplastic and neoplastic effects, respectively, indicate low health concern. Accordingly, a non-neoplastic MOE of less than 200 indicates that consumption of tarhana, a cereal-based product, was associated with health concerns, while a neoplastic MOE of less than 10,000 indicates that the MOE contribution of tarhana was of concern.

Kabak [27] pointed out that the average OTA intake of the Turkish population with respect to consumption of retail cereal products is 0.032 ng/kg b.w./day for adults and 0.097 ng/kg b.w./day for children. It was stated that these daily intake values are substantially below the JECFA estimated daily intake value of 14 ng/kg b.w./day; therefore, OTA exposure due to the consumption of retail cereal products would not pose a health risk for adults and children in Türkiye, in agreement with the present study. Hoteit et al. [65] stated that the mean OTA level detected in cereals and cereal products was 0.843 ± 1.98 μg/kg and emphasized that this value was below the limit value set by the EC. Additionally, the authors determined the OTA exposure due to the consumption of cereals and cereal products to be 3.29 ng/kg b.w./day, which is lower than the PTWI of 100 ng/kg b.w./day as determined by JECFA. The non-neoplastic MOE value (1154.45) was greater than 200, while the neoplastic MOE (3539) was below 10,000, which was found to be alarming, and cereals were regarded as foods contributing to this high MOE by the researchers.

Han et al. [66] detected OTA in 21% (21/100) of cereals and cereal-derived products, with a mean value of 0.94 ± 1.48 μg/kg in China. The dietary intake of OTA was calculated to be 1.093 ng/kg b.w./day with the consumption of cereals and cereal-derived products, which was much lower than the tolerable weekly intake of 100 ng/kg b.w./week. Among the four groups (cereals and derivatives, beans and derivatives, dried fruits and derivatives, and grapes and derivatives), the cereals and cereal-derived products group was the one with the highest health risk through dietary OTA intake. While the risk of OTA was found to be generally low, it was also emphasized that the long-term consumption of a single food group may cause potential health risks in some cases. In European countries, exposure to OTA through the consumption of cereals and cereal-based products for adults has been determined to range from 0.06 ng/kg b.w./day (Italy) to 1.28 ng/kg b.w./day (Netherlands) [67].

Considering the mold counts, none of the industrially produced tarhana samples exceeded 10^6^ CFU/g, while 12 out of 200 homemade tarhana samples exceeded 10^6^ CFU/g and 10 out of 200 homemade tarhana samples exceeded 10^7^ CFU/g. The rate of mold counts above 10^6^ CFU/g was determined to be 6.29% (Table 5). The mold contamination in tarhana samples analyzed in the present study was in the range of 1.079–8.623 log CFU/g, with a mean value of 2.742 ± 0.84 and 3.756 ± 1.66 log CFU/g for industrially produced and homemade tarhana samples, respectively (Table 6).

Colak et al. [68] detected mold contamination in all 138 tarhana samples with the level ranged from 1.4 × 10^1^ to 5.8 × 10^7^ CFU/g. Similarly, Demirci et al. [69] reported the count of yeast and mold in the experimentally produced tarhana samples to be 7.45 ± 0.02 log CFU/g and stated that this count increased as the fermentation process progressed, especially on the first day. In a study comparing the microbiological properties of wet tarhana dough and powdered dry tarhana, the highest count of yeast and mold was determined to be 2.2 × 10^6^ CFU/g for wet dough tarhana samples [70]. Sengun and Karapınar [71] reported the mold–yeast count of tarhana samples produced with different ingredients specific to various regions in Türkiye as being between 4.5 × 10^1^ CFU/g and 1.4 × 10^3^ CFU/g. The researchers emphasized that the microbiological quality of ingredients such as spices and peppers used in tarhana production may also affect the quality of tarhana. In particular, they stated that the sun drying process of spices increases the risk of microbial contamination.

In the macroscopic evaluation of mold colonies isolated from tarhana samples in the present study, the predominant species were determined to be *Aspergillus* spp. and *Penicillium* spp. Different mold species are responsible for the production of different mycotoxins. *Penicillium verrucosum* and *Aspergillus ochraceus* are mainly responsible for OTA formation [72]. Gong et al. [73] reported that *Talaromyces*, *Fusarium*, *Aspergillus*, and *Penicillium* were the dominant genera in wheat flour among the detected foods. The researchers have indicated that mycotoxins were present in some samples, although no OTA-producing molds were isolated. They associated this finding with the hypothesis that mycotoxin contamination may be related to the raw material, as the occurrence of mycotoxins in the samples was attributed to contamination in the raw material, rather than to mold activity during the storage process. In support of this statement, Ben Miri et al. [10] noted that the occurrence of mycotoxins in food is not directly linked to the mycotoxin-producing fungi present in that food. From this point of view, it would not be a correct approach to consider correlating the presence of OTA detected in tarhana samples in our study with the species responsible for mold contamination as a primary factor. Santos et al. [74] reported that fungi belonging to *Penicillium* and *Aspergillus* genus, which can grow at low water activity values, were dominant in flour samples used as raw materials. The authors revealed that most of the fungal species isolated from the raw materials (flours) were also isolated from the processing environment, including air, and emphasized that raw materials and ambient air are important sources of contamination for the processing area. Furthermore, Tabarani et al. [24] pointed out that according to recent reports, species such as *A. niger aggregate* and *A. carbonarius* have been indicated as sources of OTA in dry foods, especially cereals.

Cereal grains are exposed to a certain level of microbial contamination depending on factors such as the water, soil, insects, manure, and animal feces to which they are exposed during the growing process. This microbial load of cereals is a critical point during their storage and processing into products, where high humidity (13% is considered to be the maximum value for short-term storage of cereals) can lead to the growth of microorganisms. In addition to the humidity level, temperature and oxygen concentration also have an influence on the variation of the microbial load. Therefore, the microflora of cereals after harvesting and storage conditions seem to be decisive for the development of microorganism species [75,76].

Molds have been shown to be the main cause of spoilage in stored cereals, cereal grains, and cereal products. In addition to the losses caused by molds to the product, some mycotoxigenic fungi species can produce mycotoxins. Mycotoxin production is in question due to the growth of mold species, especially in cereals stored under high-humidity conditions. Each mold species has a range of relative humidity and temperature values at which it can grow [72].

The pH, water activity, and moisture content of tarhana samples are given in Table 7. The pH values of industrially produced tarhana samples vary between 4.28 are 4.89, while these value was determined to be between 3.97 and 5.48 in the homemade tarhana samples. The aw values of tarhana samples range from 0.475 to 0.588 and from 0.526 to 0.769 for industrially produced and homemade tarhana samples, respectively. As the average moisture content of industrially produced (7.54%) and homemade (9.65%) tarhana samples were found to be below the limit value (10%) recommended for the moisture content of tarhana by Turkish Standards Institute [77], 72 out of 200 homemade tarhana samples were above (>10–13.36) the specified moisture content limit.

Several factors affecting the growth and mycotoxin production of mycotoxigenic fungi species responsible for OTA formation are based on the water activity of cereal grains and the temperature and humidity to which the grains are exposed during storage. The initial water activity (moisture content) of harvested grains is critical to preventing fungal growth and toxinogenesis during storage. In this sense, at water activity values below 0.65, the growth of fungi is suppressed, and the germination of spores is prevented. Water activity values above 0.80, moisture content of more than 16%, and temperatures between 25 °C and 30 °C were indicated as the optimum climate conditions for mycotoxin formation [78,79]. An increase in the moisture content of the product may cause the growth of mold, which may lead to the formation of mycotoxins. Accordingly, the Turkish Standards Institute recommended that the moisture content of tarhana samples be no more than 10% [TSI, 2004].

Hassan et al. [80] proposed that mold growth and subsequent mycotoxin production may be suppressed by decreasing the moisture content of the packaged product during storage and transport. They also related the presence of molds and mycotoxin formation in the products to the countries where the products were grown/harvested. They asserted that hot and humid weather conditions in Asian countries support mold production and mycotoxin formation.

The correlations between the OTA levels in the analyzed tarhana samples and the mold counts and physicochemical parameters of these samples was evaluated using Pearson correlation coefficients (Table 8). There was a very high positive correlation between the OTA level and the mold counts (r = 0.942; *p* < 0.01) and between the OTA level and the detection limit (r = 0.991; *p* < 0.01). Moreover, the positive correlation between the OTA level and the a_w_ was very high (r = 0.730; *p* < 0.01), as was the positive correlation between the OTA level and the moisture content (r = 0.790; *p* < 0.01). However, a weak positive correlation was found between the OTA level and the pH value (r = 0.121; *p* < 0.05).

Prevention of OTA formation in food is critical to the control of potential public health concerns. The economic losses resulting from the deleterious effects of this mycotoxin on crops is another consideration [10]. From this perspective, in order to prevent or mitigate OTA contamination in tarhana, it is necessary to focus on three main aspects: raw materials, production process, and post-production conditions. The effective implementation of good agricultural practices has a critical role in ensuring food safety in cereals, which are among the foods with the highest prevalence of OTA contamination. Inadequate pre-harvest practices may cause the contamination of cereals with fungi, especially *Aspergillus* and *Penicillium* species, and may lead to occurrence of OTA associated with fungal growth during storage of cereals.

The fermentation and drying stages during the processing of tarhana are addressed to reduce the risk of OTA contamination. In this study, the uncontrolled, open-air, and/or inadequate drying technique applied to homemade tarhana during production was one of the reasons for the high OTA levels found in this tarhana production type. In industrial production, controlled drying in specialized ovens ensures a safe manufacturing application. Moreover, processing at low temperatures and humidity is seen as a crucial practice to not encourage the growth of OTA-producing molds, which is provided in industrially produced tarhana. Another critical point is the controlled fermentation process, which is essential to preventing the growth of undesirable fungi. In particular, prolonged fermentation increases the likelihood of mold growth, which can lead to high OTA levels. Uncontrolled fermentation at this stage in homemade tarhana can lead to the development of diverse fungal flora. Third, the storage conditions applied to the final product (temperature, humidity, and packaging integrity) can positively or negatively affect the level of possible contamination during the storage process.

## 4. Conclusions

This study assessed the occurrence of OTA in tarhana, a traditional cereal-based product widely consumed in Türkiye, and the potential levels of OTA exposure through consumption of this product. The higher OTA levels in homemade tarhana samples compared to industrially produced tarhana were associated with uncontrolled process conditions during homemade production, including drying and/or fermentation stages, inappropriate storage conditions, environmental factors such as temperature and humidity, and inadequate hygiene practices. Meanwhile, the estimated weekly intake levels of the tarhana samples analyzed in this study were found to be well below the tolerable weekly intake level of 100 ng/kg b.w./week. The EWI determined for the consumption of homemade tarhana samples was higher than that for industrially produced tarhana, but the exposure to both types of tarhana samples was found to be considerably lower than the PTWI and not likely to cause a health risk. The fact that tarhana consumption may, considering the MOE contribution, be of health concern indicates that critical practices need to be introduced to prevent and/or reduce OTA contamination in tarhana. The selection of raw materials to be used in tarhana production, processing steps, especially fermentation, and drying requirements and storage conditions are the indispensable steps that must be carried out regardfully. Furthermore, measures such as using fungus-resistant ingredient types, optimizing drying conditions, and ensuring proper hygiene during production can help to reduce the risk of OTA contamination. Furthermore, the regular monitoring and testing of OTA levels in tarhana products will help to ensure food safety. In conclusion, while the occurrence of OTA in tarhana is of concern, the safety of this traditional food can be ensured by significantly reducing the risk of contamination through good production and hygiene practices and quality control protocols.

## Figures and Tables

**Table 1:** Occurrence of OTA and distribution levels in tarhana samples (*n* = 350).

Detected Level (μg/kg)	Industrially Produced Tarhana (*n* = 36/150)	Homemade Tarhana (*n* = 118/200)	Total Tarhana (*n* = 154/350)	Mean ± SD of Total Tarhana Samples (μg/kg)
ND * (<0.10)	114 (76%)	82 (41%)	196 (56%)	-
0.10–0.50	15 (10%)	16 (8%)	31 (8.86%)	0.314 ^g^ ± 0.115
0.51–1.0	7 (4.67%)	18 (9%)	25 (7.14%)	0.773 ^f^ ± 0.144
1.01–1.50	9 (6%)	31 (15.5%)	40 (11.43%)	1.340 ^e^ ± 0.122
1.51–2.0	3 (2%)	24 (12%)	27 (7.71%)	1.792 ^d^ ± 0.146
2.01–2.5	2 (1.33%)	12 (6%)	14 (4%)	2.320 ^c^ ± 0.126
2.51–3.0	-	9 (4.5%)	9 (2.57%)	2.751 ^b^ ± 0.143
>3.0 ^a^	-	8 (4%)	8 (2.29%)	3.646 ^a^ ± 0.287

* ND: not detected (detection limit: 0.10 μg/kg); SD: standard deviation. ^a^: The maximum permissible limit of OTA set by the Turkish Food Codex [8] and European Commission [9]. ^a–g^: Means within a column with different letters are significantly different (*p* < 0.001).

**Table 2:** Mean and range values of OTA in homemade and industrially produced tarhana samples (*n* = 350).

Sample	*n*	*n* ^p^	Min (μg/kg)	Max (μg/kg)	Mean ± SD (μg/kg)	CI_95_ (%)	>3.0 μg/kg (%)
Industrially produced tarhana	150	36	0.12	2.34	0.21 ± 0.64	0.108–0.312	-
Homemade tarhana	200	118	0.16	4.15	0.93 ± 0.90	0.805–1.055	8 (4)
Total tarhana	350	154	0.12	4.15	0.62 ± 0.92	0.576–0.716	8 (2.29)

*n*: total number of tarhana samples; *n*^p^: number of positive; SD: standard deviation; CI_95_: 95% confidence interval.

**Table 3:** The dietary intake (estimated weekly intake) of OTA from consumption of tarhana samples.

Sample	EWI				PTWI (ng/kg b.w./Week)	PTWI (ng/70 kg b.w./Week)	% PTWI			
	GROUP 1 (1 Time/Week)	GROUP 2 (3 Times/Week)	GROUP 3 (5 Times/Week)	GROUP 4 (7 Times/Week)			GROUP 1 (1 Time/Week)	GROUP 2 (3 Times/Week)	GROUP 3 (5 Times/Week)	GROUP 4 (7 Times/Week)
Industrially produced tarhana	0.06	0.18	0.30	0.42	100	7000	0.1	0.2	0.3	0.4
Homemade tarhana	0.27	0.80	1.33	1.86	100	7000	0.3	0.8	1.3	1.9

EWI: estimated weekly intake (ng/kg b.w./week); PTWI: provisional tolerable weekly intake of OTA set by JECFA (ng/kg b.w./week); b.w.: tarhana consumed on one occasion is assumed to be 20 g.

**Table 4:** Estimated daily intake of OTA and margin of exposure (MOE) for average and extreme intake of homemade and industrially produced tarhana.

Sample	EDI		MOE ^a^		MOE ^b^	
	Average Intake (1 Portion/Day)	Extreme Intake (3 Portion/Day)	Average Intake (1 Portion/Day)	Extreme Intake (3 Portion/Day)	Average Intake (1 Portion/Day)	Extreme Intake (3 Portion/Day)
Industrially produced tarhana	0.06	0.18	78.83	26.28	241.67	80.56
Homemade tarhana	0.27	0.80	17.78	5.94	54.51	18.19

EDI: estimated daily intake (ng/kg b.w./week); MOE: margin of exposure. ^a^: non-neoplastic effect; ^b^: neoplastic effect; 1 portion: equal to 20 g tarhana consumption.

**Table 5:** Distribution of mold contamination in tarhana samples (*n* = 350).

Sample	*n*	*n* ^p^	ND (<10)	10–<10^2^	10^2^–<10^3^	10^3^–<10^4^	10^4^–<10^5^	10^5^–<10^6^	10^6^–<10^7^	>10^7^
Industrially produced tarhana	150	Number	-	60	44	29	12	5	-	-
		Rate (%)	-	40	29.33	19.33	8	3.33	-	-
Homemade tarhana	200	Number	-	38	54	41	32	13	12	10
		Rate (%)	-	19	27	20.5	16	6.5	6	5
Total tarhana	350	Number	-	98	98	70	44	18	12	10
		Rate (%)	-	28	28	20	12.57	5.14	3.43	2.86

**Table 6:** Mean and range values of mold counts detected in homemade and industrially produced tarhana samples (*n* = 350).

Sample	*n*	Min (log CFU/g)	Max (log CFU/g)	Mean ± SD (log CFU/g)
Industrially produced tarhana	150	1.079	6.394	2.742 ± 0.84
Homemade tarhana	200	1.477	8.623	3.756 ± 1.66
Total tarhana	350	1.079	8.623	3.321 ± 1.55

**Table 7:** pH, water activity, and moisture content values of tarhana samples (*n* = 350).

Parameter	Sample	*n*	Min	Max	Average
pH	Industrially produced tarhana	150	4.28	4.89	4.565
	Homemade tarhana	200	3.97	5.48	4.707
	Total tarhana	350	3.97	5.48	4.646
a_w_	Industrially produced tarhana	150	0.475	0.588	0.504
	Homemade tarhana	200	0.526	0.769	0.593
	Total tarhana	350	0.475	0.769	0.555
Moisture content (%)	Industrially produced tarhana	150	6.42	8.97	7.535
	Homemade tarhana	200	7.89	13.36	9.646
	Total tarhana	350	6.42	13.36	8.741

**Table 8:** Correlation coefficients (r) among the OTA levels, mold counts, and physicochemical parameters of tarhana samples.

Parameters	Group	Detection Limit	OTA	Mold	pH	a_w_	Moisture Content
Group	1	0.393 **	0.391 **	0.324 **	0.262 **	0.733 **	0.669 **
Detection Limit		1	0.991 **	0.940 **	0.107 *	0.724 **	0.791 **
OTA			1	0.942 **	0.121 *	0.730 **	0.790 **
Mold				1	0.137 *	0.693 **	0.806 **
pH					1	0.279 **	0.182 **
a_w_						1	0.799 **
Moisture content							1

**: Correlation is significant at the 0.01 level (two-tailed). *: Correlation is significant at the 0.05 level (two-tailed).

## Data Availability

The original contributions presented in this study are included in the article. Further inquiries can be directed to the corresponding author.
